# Difference and environmental drivers of bacterial communities on wall paintings of the Maijishan and Mogao Grottoes, China

**DOI:** 10.3389/fmicb.2025.1657118

**Published:** 2025-09-24

**Authors:** Wenxia Ma, Qiqi Chen, Fasi Wu, Dongpeng He, Yulong Duan, Yongqiang Yue, Ji-Dong Gu, Xiaoyan Yang, Huyuan Feng

**Affiliations:** ^1^Key Laboratory of Western China’s Environmental Systems (Ministry of Education), Key Scientific Research Base of Bioarchaeology in Cold and Arid Regions (National Cultural Heritage Administration), College of Earth and Environmental Sciences, Lanzhou University, Lanzhou, Gansu, China; ^2^MOE Key Laboratory of Cell Activities and Stress Adaptations, School of Life Sciences, Lanzhou University, Lanzhou, Gansu, China; ^3^National Research Center for Conservation of Ancient Wall Paintings and Earthen Sites, Conservation Institute, Dunhuang Academy, Dunhuang, Gansu, China; ^4^Key Laboratory of Extreme Environmental Microbial Resources and Engineering, Gansu Province, Northwest Institute of Eco-Environment and Resources, Chinese Academy of Sciences, Lanzhou, Gansu, China; ^5^Institute of Maijishan Grottoes Art, Dunhuang Academy, Tianshui, Gansu, China; ^6^Environmental Science and Engineering Group, Guangdong Technion-Israel Institute of Technology, Shantou, Guangdong, China; ^7^Guangdong Provincial Key Laboratory of Materials and Technologies for Energy Conversion, Guangdong Technion-Israel Institute of Technology, Shantou, Guangdong, China

**Keywords:** microbial damages, DNA-RNA high-throughput sequencing, relative humidity, environmental monitoring, sustainable preservation

## Abstract

The Maijishan and Mogao Grottoes, both UNESCO World Heritage Sites along the Silk Road, are increasingly threatened by microbial biodeterioration. To characterize bacterial communities of different microbial damages on wall paintings and identify environmental drivers, we combined high-throughput DNA/RNA sequencing with microenvironmental monitoring and conducted a cross-site comparison. At Maijishan, bacterial communities associated with black and white mycelia showed no significant compositional differences within the same cave but varied markedly between caves, indicating site-specific community assembly. Actinobacteria (>50%), particularly *Pseudonocardia* and *Actinomycetospora*, predominated, while RNA-based analysis revealed active populations of *Escherichia* and *Stenotrophomonas*, likely introduced via exogenous contamination from animal activities. In contrast, black spots from the Mogao Grottoes were dominated by Actinobacteria, Firmicutes, and Proteobacteria, with *Rhodococcus* as a core genus. No core bacterial OTUs were shared between the sites, suggesting strong microenvironmental filtering. Multivariate analysis identified substrate properties (total organic carbon, total nitrogen, pH) and microclimatic fluctuations (diurnal temperature/humidity ranges) as critical drivers. Maijishan’s persistently humid conditions (RH > 70% for over 180 days/ year) favored Actinobacteria proliferation, whereas Mogao’s arid climate (RH < 70% for over 240 days/year) selected for xerotolerant Firmicutes. These results reveal distinct site-specific microbial colonization patterns and provide a scientific basis for targeted conservation strategies to mitigate microbial damage and preserve these invaluable wall paintings.

## 1 Introduction

Wall paintings represent one of the most ancient and enduring expressive forms of expression human creativity, bearing exceptional artistic, scientific, and historical significance on a global scale. As prominent components of grotto architecture, they provide rich insights into the evolution of societies and civilizations (Garg et al., 1995; Sansupa et al., 2023; Wu et al., 2022). However, these cultural assets face increasing threats by biodeterioration, a process exacerbated by global climate change characterized by rising temperature variability and intensified hydrological extremes (Leissner et al., 2015; Liu et al., 2020; Ding et al., 2022; Hu and Hewitt, 2024). Among the various degradation agents, microbial activity plays a central role, driving damage through both physicochemical and biochemical mechanisms. Bacteria, in particular, are often the pioneering colonizers on wall-painting surfaces (Cuezva et al., 2012; Ma et al., 2015; Meng et al., 2017; Mihajlovski et al., 2017; Sugiyama et al., 2017; Ma C. et al., 2023). Their metabolites, such as organic acids, pigments and extracellular polymeric substances (EPS), which accelerate the aesthetic value and structural integrity reducing (Abdel-Haliem et al., 2013; Cojoc et al., 2019; Dominguez-Moñino et al., 2017; Elhagrassy, 2018). Through photoautotrophic or chemoautotrophic metabolism, bacterial communities fix carbon and generate organic substrates, facilitating the succession of heterotrophic fungi or archaea (Lan et al., 2010; Li et al., 2021; Liu et al., 2020; Zhang et al., 2019). This trophic cascade fundamentally reshapes microhabitat conditions, promoting secondary colonization by other microorganisms (Xu et al., 2018).

Biodeterioration poses major challenges to wall painting preservation, understanding these microbial communities is thus essential for heritage conservation (Ciferri, 1999; Martin-Sanchez et al., 2012; Ma et al., 2020; Pei et al., 2023). While previous research has relied heavily on culture-dependent methods or DNA-based high-throughput sequencing to characterize microbial diversity, these approaches have limitations. DNA sequencing captures the total community composition, including dormant or dead cells, but does not directly reveal which members are metabolically active. RNA enables the identification of taxa actively participating in biodeterioration at the sampling moment, the integration of DNA and RNA sequencing provides a more complete and dynamic view of microbial communities by resolving both the taxonomic structure (DNA) and the metabolically active fraction (RNA). Applying such an integrated method significantly enhances our ability to investigate the viability of these microbial communities and allows more accurate assessment of microbial threats, thereby improving the scientific basis for preventive conservation (Sanmartín et al., 2018; Meng et al., 2020; Liu W. et al., 2022).

Microbial communities colonizing cultural heritage materials are shaped by a complex interplay of environmental conditions, biological and physicochemical factors (Dornieden et al., 2000; Wu et al., 2021; Zhang et al., 2021). Previous studies have demonstrated that regional climate type and microclimatic fluctuations are key determinants of microbial diversity in heritage sites (Ding et al., 2022), with temperature and precipitation patterns identified as key drivers of microbial diversity (Biagioli et al., 2024; Chen and Gu, 2022; Liu et al., 2018). Bacterial communities, in particular, tend to respond more rapidly and sensitively to environmental variability than fungi, which often display greater adaptability and resilience (Barnard et al., 2013; Chen et al., 2023). This sensitivity means that climate-induced environmental shifts can trigger rapid changes in bacterial assemblages, potentially leading to irreversible deterioration trajectories (Viles and Cutler, 2012; Li et al., 2021; Ding et al., 2022).

This study investigates microbial biodeterioration at two UNESCO World Heritage Sites along the Ancient Silk Road: the Maijishan Grottoes and the Mogao Grottoes. In recent years, microbial proliferations have been observed in Caves No. 28 and No. 30 at Maijishan, appearing as black and white mycelial growths (Ma et al., 2025). While black spot formations have previously been documented in Cave No. 256 at Mogao (Ma W. et al., 2023). By comparing the bacterial communities and microenvironmental conditions across these geographically distinct sites (separated by over 1,400 km), this study aims to: (1) elucidate taxonomic divergences between black and white mycelia in Maijishan Grottoes; (2) identify site-specific bacterial community signatures distinguishing the two sites; and (3) evaluate the relative influence of geographic isolation versus environmental filtering on bacterial community assembly. These findings aim to advance our understanding of microbial biogeography in wall painting environments and provide a scientific foundation for developing site-specific conservation strategies.

## 2 Materials and methods

### 2.1 Description of study sites

**Figure 1:**
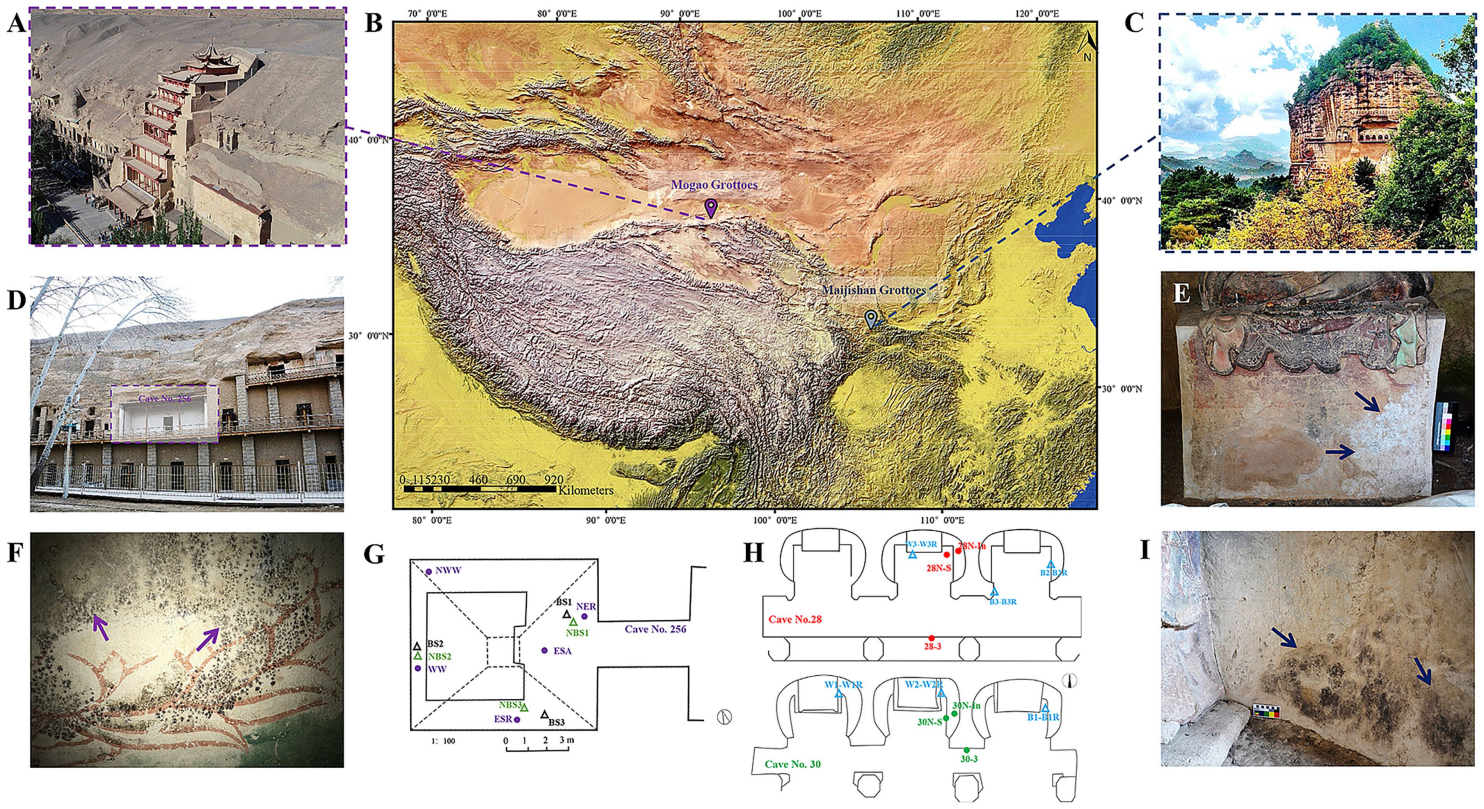
Locations of the study site and typical microbial damages of the Maijishan Grottoes and the Mogao Grottoes in China. **(A–C)** The geographical locations and exterior illustration of the Maijishan and Mogao Grottoes. **(D,F)** The appearance of Cave No. 256 and black spots on wall paintings at the Mogao Grottoes. **(G)** Schematic diagram of sampling and environmental monitoring sites in Cave No. 256, the black and green triangles represent the sampling sites for black-spots samples (BS) and no-spots samples (NBS), respectively, while the purple dots represent the temperature and relative humidity monitoring sites. **(E,I)** White and black mycelium on the wall paintings of Caves No. 28 and No. 30 at the Maijishan Grottoes. **(H)** Schematic diagram of sampling and environmental monitoring sites, the blue triangles represent the sampling sites, while the red and green dots represent the temperature and relative humidity monitoring sites.

The Maijishan Grottoes (34°05′–35°10′N, 104°35′–106°44′E), a UNESCO World Cultural Heritage Site, are located in the Tianshui Maiji Mountain, a part of Qinling Mountain range. This cultural site comprises 221 caves, featuring 10,632 clay sculptures and approximately 1,000 m^2^ of wall paintings. Situated in a transitional zone between subtropical and warm temperate climates, the region is characterized by dense forest coverage, high humidity and stable temperatures year-round. In contrast, the Mogao Grottoes (39°53′–41°35′N, 92°13′–95°30′E), also designated as a UNESCO World Cultural Heritage Site, are positioned along the eastern cliff of Mingsha Mountain, approximately 25 km southeast of Dunhuang. This site includes 735 caves, with over 45,000 m^2^ of wall paintings and 2,400 sculptures, and is located in a cold desert climate zone marked by low humidity, minimal precipitation, and high potential evaporation (Figures 1A–C).

Both grotto complexes were constructed during the 4th century CE (Maijishan: 384 CE; Mogao: 366 CE), with similar wall painting techniques and materials. These include mineral pigments, natural sediments, original binders (e.g., casein, animal glue, gelatin), and plant fibers (e.g., hemp, wheat straw, cotton) used for structural reinforcement. Microbial colonization and biodeterioration have been observed to varying degrees in both the Maijishan Grottoes and the Mogao Grottoes. This study focused on Cave No. 256 at the Mogao Grottoes, where black spot deterioration is present (Figures 1D,F,G), and Caves No. 28 and No. 30 at the Maijishan Grottoes, which exhibit black and white mycelial growth on wall paintings (Figures 1E,H,I).

### 2.2 Sampling

Caves No. 28 and No. 30, situated within the middle-lower sections of the Maijishan Grottoes (Supplementary Figures S1B,C,F), with black (Supplementary Figures S1A,D,I) and white (Supplementary Figures S1E,G,H) mycelia colonizing wall paintings. In August 2021, a total of 12 microbial samples (each pair consisting of two samples) were collected from colonized wall painting surfaces. Each sample was collected using sterile forceps within a targeting 10 × 10 cm area (Supplementary Figures S1A,D,E,G–I). Each collected mycelia sample was thoroughly mixed and equally divided: one portion for DNA extraction (designated W1–W3 for white and B1–B3 for black mycelia), and the other preserved in RNAlater (Qiagen, Germany) for RNA analysis (designated W1R-W3R and B1R-B3R) to assess viable metabolism. Sampling involved carefully removing only mycelia without damage to paintings or restored materials, with each weighed approximately 50 mg. Additionally, fragments of non-colonized wall paintings (totaling 10–15 g) were collected from both caves for physicochemical characterization. All samples were stored in sterile Eppendorf tubes and transported to the laboratory at Lanzhou University for further analysis. The sampling was conducted under the authorization and supervision of professionals from the Maijishan Grottoes Art Research Institute.

For comparative analysis, previously collected and sequenced samples from Cave No. 256 at the Mogao Grottoes (Ma W. et al., 2023) were re-analyzed, including black-spot samples (BS) and non-spot (NBS) samples. These samples collected from the wall painting surfaces with the same sampling method, with specific sample information and detailed data provided in the previous publication (Ma W. et al., 2023).

### 2.3 DNA extraction, RNA extraction, and MiSeq high-throughput sequencing analysis

Total genomic DNA and rRNA from each sample were extracted using the PowerSoil^®^ DNA Isolation Kit and PowerSoil^®^ RNA Kit (MO BIO Laboratories, United States), following the manufacturer’s protocols. RNA was subsequently reverse-transcribed into complementary DNA (cDNA) with the cDNA Synthesis Kit (Sigma-Aldrich, United States). Nucleic acid concentrations (DNA/cDNA) were quantified using a NanoDrop^®^ ND-2000 spectrophotometer (Thermo Scientific, United States), and integrity was verified by 1% agarose gel electrophoresis. The bacterial V3–V4 regions of the 16S rRNA gene were amplified using primers 338F/806R (Peiffer et al., 2013). PCR conditions consisted of initial denaturation at 95°C for 3 min, followed by 35 cycles of 94°C for 30 s, 55°C for 30 s, and 72°C for 45 s, with a final extension at 72°C for 10 min (detailed reaction mixtures in Supplementary Table S1). Triplicate amplifications per sample were pooled, purified with a Gel Extraction Kit (AXYGEN, China), and quantified via QuantiFluor™-ST Fluorimeter (Promega, United States). Libraries were prepared using the NEXTflex^®^ Rapid DNA-Seq Kit (Bioo Scientific, United States) and sequenced on an Illumina MiSeq PE300 platform (Majorbio Bio-Pharm Technology Co., China).

Raw FASTQ data were processed with Trimmomatic (Bolger et al., 2014) and FLASH (Magoč and Salzberg, 2011), with reads <50 bp discarded and no assembly performed. High-quality sequences were clustered into operational taxonomic units (OTUs) at 97% similarity via UPARSE (Edgar, 2013). Taxonomic classification of bacterial OTUs was performed using the RDP Classifier against the SILVA and UNITE v7.0 databases with a 70% confidence threshold.

### 2.4 Environmental data collection

Approximately 3 g of homogenized wall painting substrate from the Maijishan Grottoes was oven-dried at 105°C until constant weight to determine gravimetric moisture content (MC). pH and electrical conductivity (EC) were measured using a Sartorius PB-10 pH meter (1:5 w/v in deionized water) and Leici DDSJ-318 conductivity meter (1:5 w/v in 1 M KCl), respectively. Total organic carbon (TOC) and total nitrogen (N) were quantified by high-temperature combustion (450°C and 1,250°C) using a Vario EL cube CHNS analyzer.

Continuous environmental monitoring was conducted from 2021 to 2022 in Caves No. 28 and No. 30 (Figure 1H). HOBO^®^ U23-001 loggers recorded air (28-3, 30-3) and wall surface (28 N-S, 30 N-S) temperature (T) and relative humidity (RH) at hourly intervals. iButton^®^ DS1923 sensors embedded in wall paintings measured internal T and RH every 2 h (28 N-IN, 30 N-IN). The environmental monitoring protocol in Cave No. 256 at the Mogao Grottoes followed the same design as detailed in our previous study (Ma W. et al., 2023), with monitoring locations shown in Figure 1G.

### 2.5 Statistical analysis

All statistical analyses were performed using SPSS 13.0. The *α*-diversity indices (Shannon, Simpson, Ace, and Chao) were calculated with MOTHUR (version 1.30.1) (Schloss et al., 2009). Redundancy analysis (RDA) was conducted in R (v4.0.3) using the vegan package to explore relationships between microbial communities and environmental variables. Sankey diagrams were generated with the ggalluvial package within the R environment. Differences between sample groups were tested with the Wilcoxon rank-sum test (*p* < 0.05). Environmental parameter visualizations were created using Origin 8.0 software.

## 3 Results and analysis

### 3.1 Bacterial community diversity and taxonomic composition in Maijishan Grottoes

**Figure 2:**
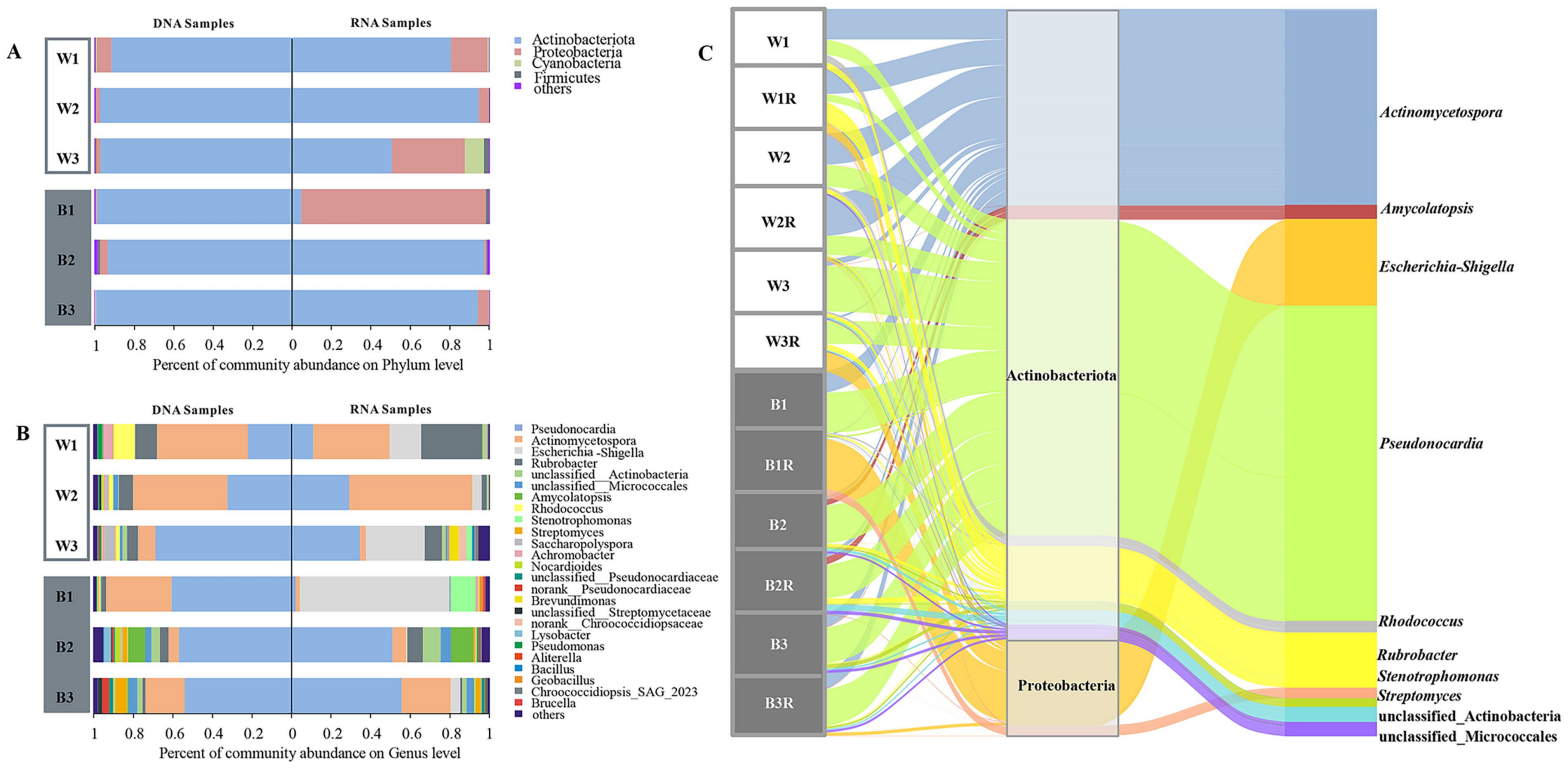
Community structures of bacteria detected at DNA and RNA levels. **(A,B)** Bacterial phyla and genera. **(C)** The Sankey diagram of bacterial microbial community structures based on the 16S rRNA gene at DNA and RNA levels.

High-throughput sequencing of the bacterial V3–V4 regions from both DNA and cDNA libraries yielded 782,304 raw sequences, clustered into 526 OTUs, with the specific sequence counts, OTU numbers, and diversity indices for each sample presented in the Supplementary Table S2. Alpha-diversity metrics revealed minimal differences among the four sampling types (Black-DNA, Black-RNA, White-DNA, White-RNA, *p* < 0.05, Wilcoxon rank-sum test), with the Black-RNA group exhibiting the highest Shannon index, followed by Black-DNA, White-DNA, and White-RNA (Supplementary Table S2). Taxonomic classification identified 21 bacterial phyla, 182 families, and 302 genera. The dominant phyla were Actinobacteria, Proteobacteria, Cyanobacteria, and Firmicutes, with Actinobacteria exceeding 50% relative abundance in all but one RNA sample (B1R). RNA samples showed higher proportions of Proteobacteria, suggesting elevated microbial viability (Figure 2A).

At the genus level, *Pseudonocardia* and *Actinomycetospora* dominated, accounting for >60% relative abundance in most samples (excluding W3R and B1R, Figure 2B). *Pseudonocardia* was predominant in black mycelial and some white mycelial samples (B1, B2, B2R, B3, B3R, W3, W3R), while Actinomycetospora was more abundant in other white mycelial groups (W1, W1R, W2, W2R) (Figure 2C). Kruskal-Wallis H test revealed no significant taxonomic differences among the four groups. Among the top ten most abundant bacterial genera, *Pseudonocardia* and *Actinomycetospora* exhibited similar content in four groups. Notably, RNA samples consistently showed higher levels of *Escherichia* and *Stenotrophomonas* in bacterial taxa, indicating higher microbial viability (Supplementary Figure S2).

### 3.2 Influence of environmental factors and painting materials on bacterial communities in Maijishan Grottoes

The wall painting substrates in Caves No. 28 and No. 30 exhibited weak alkalinity (pH 8–9). Cave No. 28 showed lower moisture content and electrical conductivity than Cave No. 30 but had higher total organic carbon (TOC) and total nitrogen (N) levels (Table 1).

**Table 1:** Comparative analysis of physicochemical parameters of two caves at the Maijishan Grottoes.

Sampling cave	pH	MC (%)	EC	TOC	TN
Cave No. 28	8.380 ± 0.221	3.133	1.470 ± 0.116	1.556 ± 0.104	0.255 ± 0.017
Cave No. 30	8.166 ± 0.022	3.657	2.876 ± 0.122	1.156 ± 0.089	0.107 ± 0.032

The pH, moisture content (MC), electrical conductivity (EC), total organic carbon (TOC) and total nitrogen (TN) of Cave No. 28 and Cave No. 30 in the Maijishan Grottoes.

**Figure 3:**
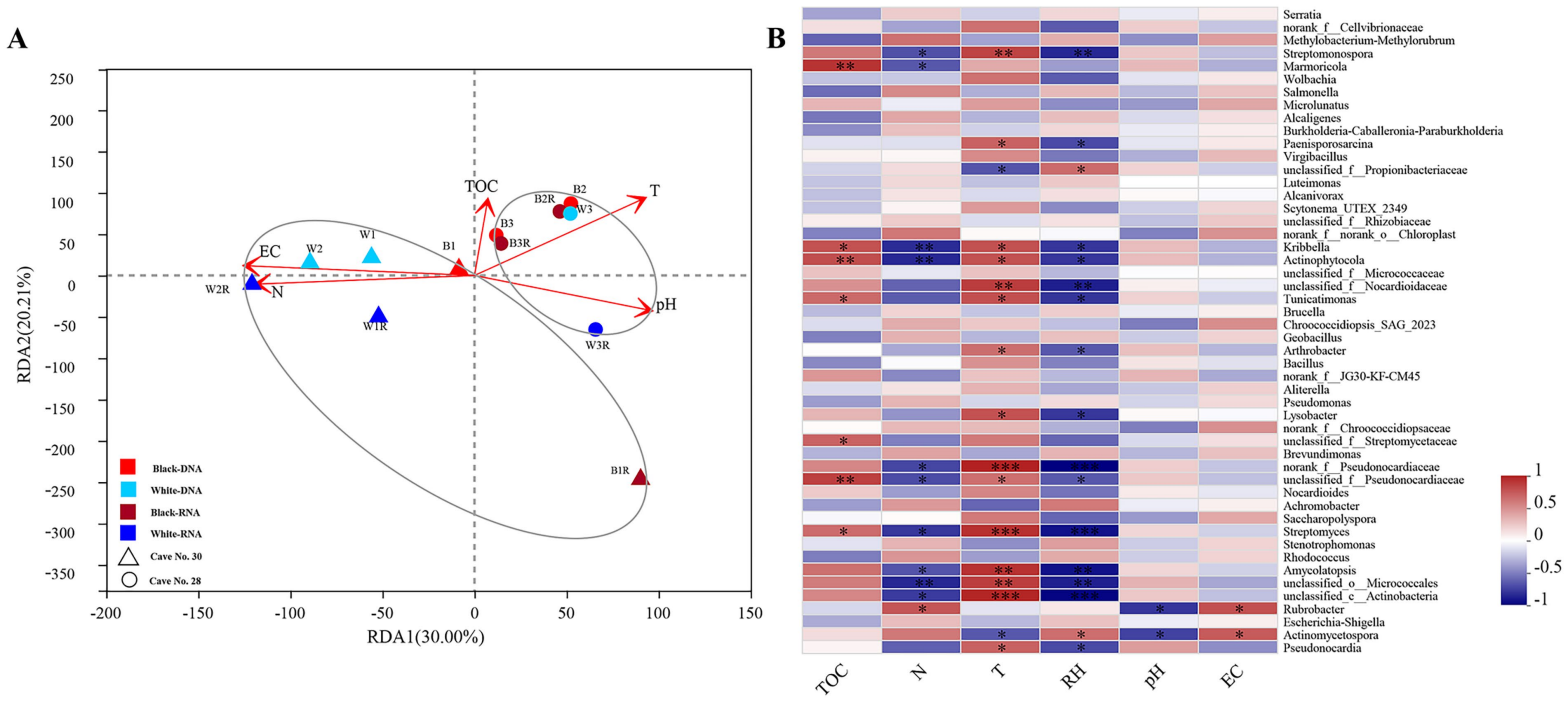
Correlation analysis between microbial community structure and environmental factors. RDA analysis on the genera level of bacteria. **(A)** Showing the correlations between the sample types and environmental factors. Spearman correlation heatmap analysis showed the associations between the top 50 dominant bacterial **(B)** genera and the environmental factors. Note: TOC-total organic carbon, N- total nitrogen, T- temperature, RH- relative humidity, EC- electrical conductivity. *: q < 0.05 (FDR-corrected); **: q < 0.01 (FDR-corrected); ***: q < 0.001(FDR-corrected). Red and dark red indicate DNA and RNA samples from black mycelia; sky blue and dark blue indicate DNA and RNA samples from white mycelia. Circles are from Cave No. 28, triangles from Cave No. 30.

To identify key environmental drivers, variance inflation factor (VIF) analysis employed to eliminate inappropriate factors (e.g., moisture content, MC) with a threshold of 10. Redundancy Analysis (RDA) was then utilized to ascertain the relationships among microbial samples, basic physicochemical characteristics, and environmental factors (Figure 3A). While black and white mycelia showed no significant compositional differences, samples clustered strongly by cave origin regardless of mycelium color. In Cave No. 28, bacterial community composition was primarily shaped by temperature and pH, whereas in Cave No. 30, total nitrogen and electrical conductivity were the dominant factors. To rigorously control the false discovery rate (FDR) in high-dimensional correlation testing, we applied Benjamini-Hochberg correction to all pairwise associations. Heatmap visualization (Figure 3B) revealed that temperature (T) and relative humidity (RH) exerted the most contrasting effects on community structure. Six Actinobacterial genera, including unclassified_c_Actinobacteria, *Streptomyces*, and norank_f__Pseudonocardiaceae taxa displayed significant positive correlations with T and negative correlations with RH (q < 0.0001, FDR-corrected), while *Streptomonospora*, unclassified_f__Nocardioidaceae, *Amycolatopsis*, and unclassified_o__Micrococcales taxa showed positive correlations with T (q < 0.05, FDR-corrected). Furthermore, total nitrogen (N) exhibited significant negative correlations with unclassified_o__Micrococcales, *Kribbella* and *Actinophytocola* (q < 0.05, FDR-corrected), whereas total organic carbon (TOC) demonstrated pronounced positive association with *Actinophytocola* (q < 0.05, FDR-corrected).

### 3.3 Comparison of bacterial composition in samples from the Maijishan and Mogao Grottoes

**Figure 4:**
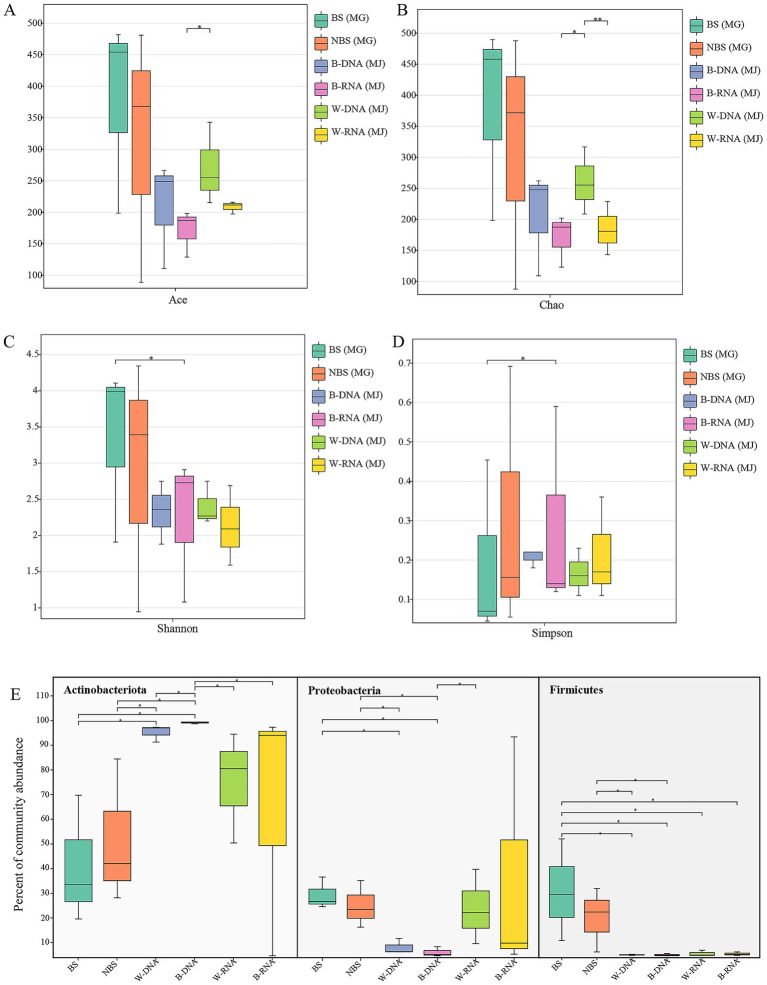
Comparison of bacterial alpha diversity and dominant bacterial phyla between the Maijishan Grottoes and Mogao Grottoes by Wilcoxon rank-sum test. **(A,B)** Species richness estimators (ACE and Chao indices), **(C,D)** alpha diversity indices (Shannon and Simpson), and dominant bacterial phyla **(E)**.

**Figure 5:**
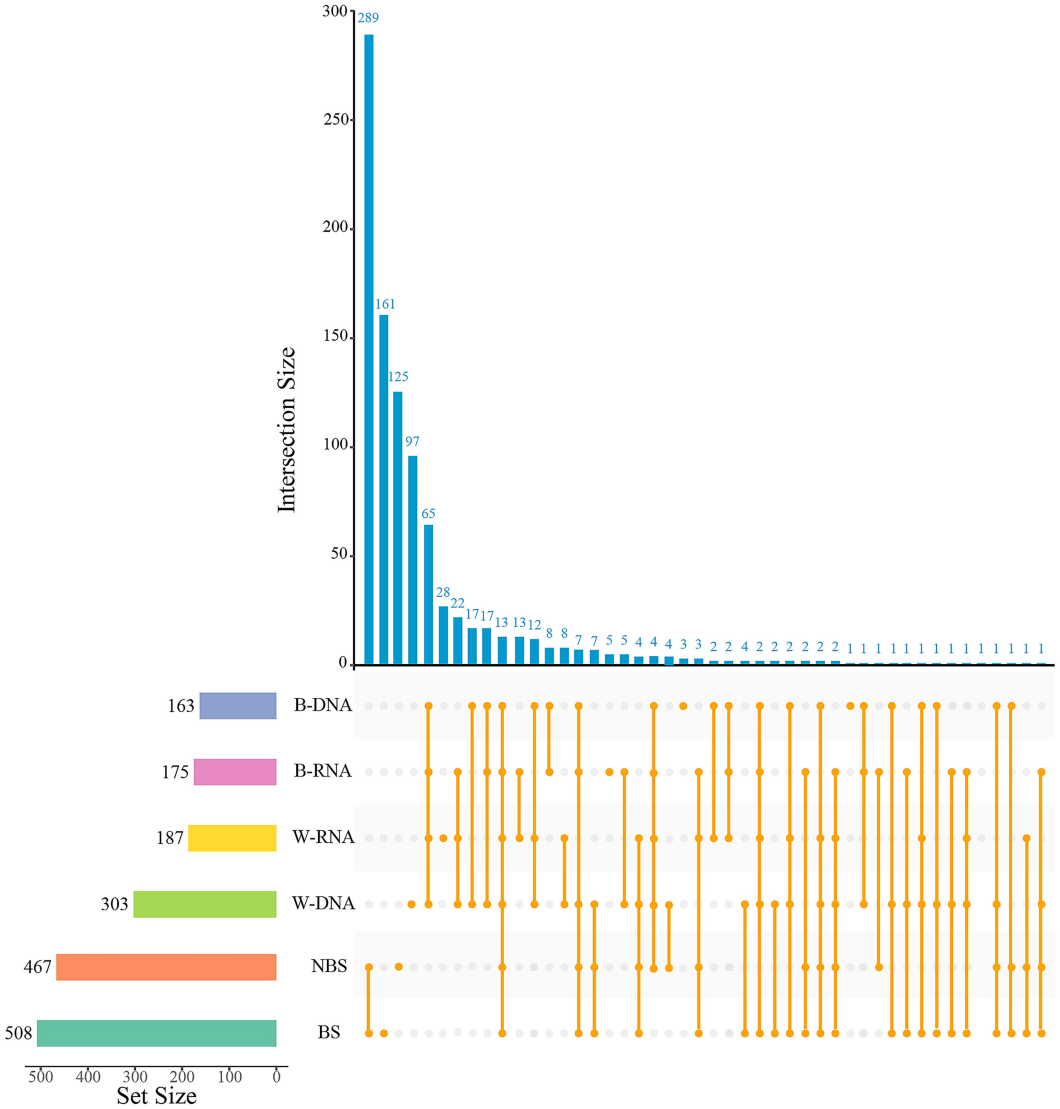
The Upset diagram showing the intersection of bacterial OTUs in different type samples from Mogao Grottoes and Maijishan Grottoes. The dark-blue histogram shows the quantity of intersection OTUs; the histogram of the left shows the total of OTUs in each sample, and the orange dots and lines represent the intersection of samples.

Comparative analysis of alpha-diversity revealed statistical differences in bacterial diversity within black microbial samples between the Maijishan Grottoes and the Mogao Grottoes (*p* < 0.05, Wilcoxon rank-sum test, Figures 4A–D). Both sites were dominated by Actinobacteria, Proteobacteria and Firmicutes, but their relative abundances exhibited site-specific patterns: Actinobacteria were more abundant in Maijishan samples, whereas Proteobacteria and Firmicutes showed higher abundance in Mogao samples (*p* < 0.05, Wilcoxon rank-sum test, Figure 4E). Upset analysis revealed that Maijishan groups (B-DNA, B-RNA, W-RNA, W-DNA) obtained 163, 175, 187, and 303 OTUs respectively, significantly fewer than the OTU numbers in Mogao samples (467 and 508 OTUs). Among these, 575 OTUs were detected only in the Mogao Grottoes samples, including 289 OTUs detected in both black spots and non-spots samples. Additionally, 209 OTUs were detected only in the Maijishan Grottoes samples, including 65 OTUs detected in both black mycelia DNA/RNA and white mycelia DNA/RNA samples (Figure 5). Only 13 OTUs were shared among the six types of samples from the two sites, none of which could be resolved at the species level, and all had low read counts.

**Figure 6:**
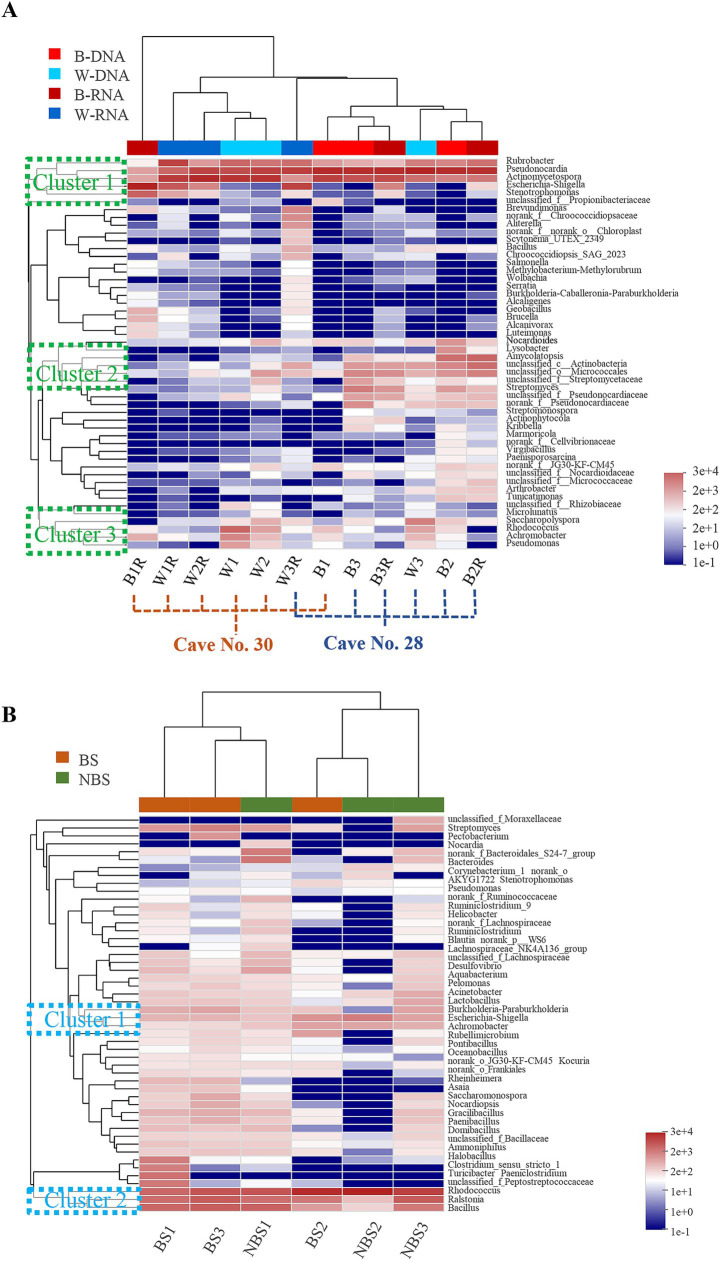
Heatmap and hierarchical cluster analysis of bacterial communities with the top 50 abundant genera in the Maijishan Grottoes **(A)** and Mogao Grottoes **(B)**, respectively.

Hierarchical clustering of the top 50 bacterial genera revealed site-specific community composition (Figure 6). Maijishan samples clustered by cave origin, one was for 5 samples collected from Cave No. 30, the other group included 6 samples collected from Cave No. 28 (Figure 6A). Genera were organized into three main clusters, Cluster 1, which included *Rubrobacter*, *Pseudonocardia*, *Actinomycetospora*, *Escherichia-Shigella*, and *Stenotrophomonas*, was predominantly present across nearly all samples. Cluster 2, comprising *Nocardioides*, *Lysobacter*, *Amycolatopsis*, etc., was primarily concentrated in samples derived from Cave No. 28. In contrast, Cluster 3, which included *Saccharopolyspora*, *Rhodococcus*, *Achromobacter*, and *Pseudomonas*, exhibited no discernible distribution pattern. In contrast, Mogao Cave No. 256 samples were divided into two main groups based on sampling locations, the samples located proximal to the tunnel passageway formed one group (BS1, BS3, and NBS1), whereas interior-positioned samples formed another (BS2, NBS2 and NBS3). Dominant genera formed two key clusters: Cluster 1 included *Escherichia-Shigella* and *Achromobacter*, Cluster 2 included *Rhodococcus*, *Ralstonia*, and *Bacillus*, distributed across various samples.

### 3.4 Comparison of diurnal variation of temperature in the Maijishan and Mogao Grottoes

In Maijishan Grottoes, mean diurnal range of air temperature (28-3, 30-3) were significantly higher than those of wall painting surfaces (28 N-S, 30 N-S) and plaster layers (28 N-IN, 30 N-IN). Within each cave system, the mean diurnal range of temperature of wall painting surfaces exceeded plaster layers, indicating that air temperature was more susceptible to external environmental disturbances. Cave No. 30 experienced the most pronounced thermal variability, with the mean diurnal range of air temperature exceeding 5°C annually and peaking at 12°C during November–December, markedly higher than equivalent periods in Cave No. 28. Both caves exhibited minimal diurnal temperature fluctuations (mean range < 2°C) across wall painting surfaces and plaster substrates. However, Cave No. 30 displayed consistently elevated temperature ranges at corresponding monitoring points relative to Cave No. 28 (Supplementary Figure S3A). Contrastingly, the Cave No. 256 of Mogao Grottoes demonstrated smaller mean diurnal range of temperature across five monitoring points. Excluding cave air (ESA), all measurement positions maintained sub-0.5°C fluctuation thresholds, the west wall (WW) exhibited the highest mean diurnal range of temperature in plaster layers for nearly half the year (January, February, August, November, and December, Supplementary Figure S3B).

### 3.5 Comparison of diurnal variation of relative humidity in Maijishan and Mogao Grottoes

**Figure 7:**
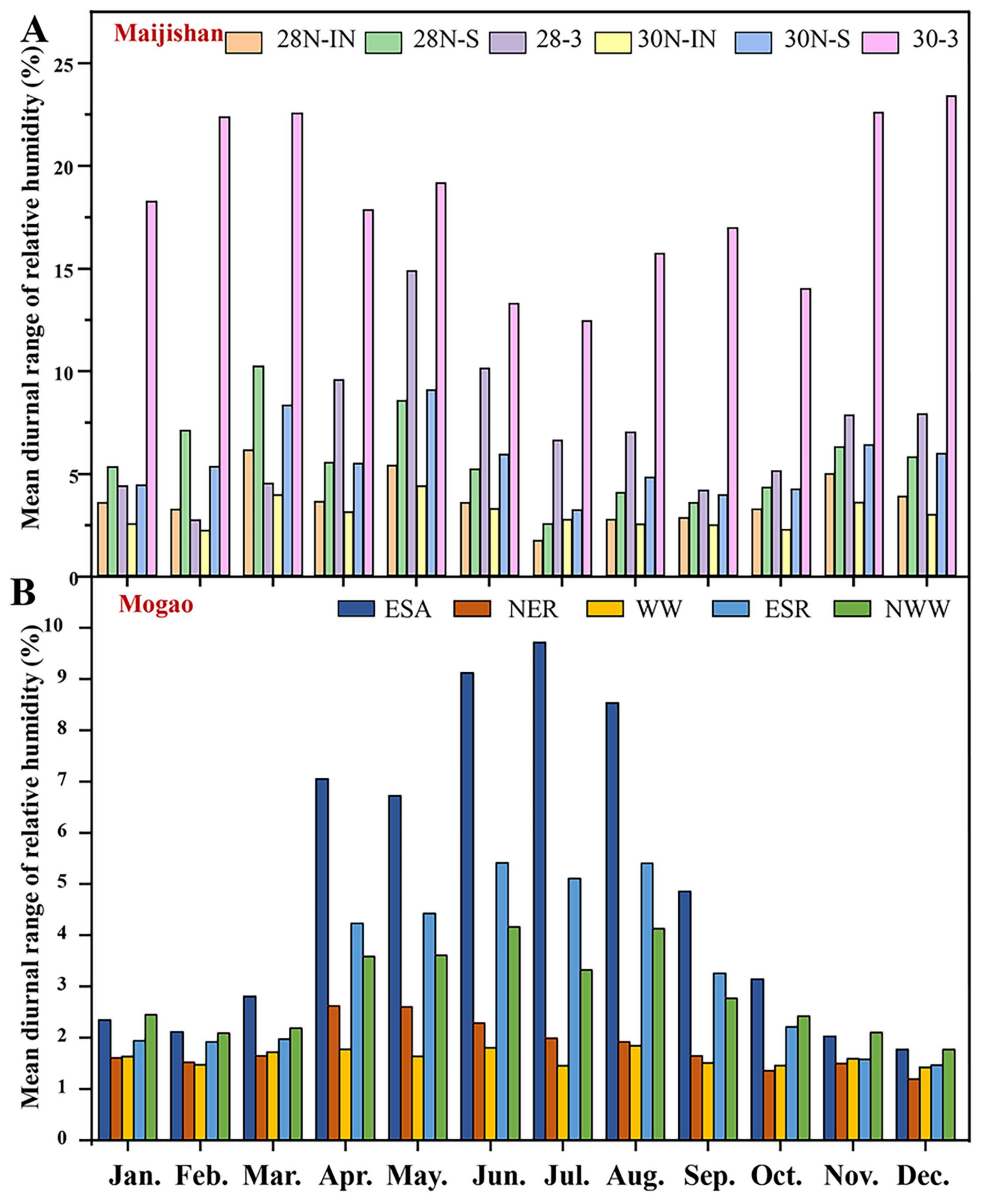
Mean diurnal range of relative humidity within Caves No. 28 and No. 30 of the Maijishan Grottoes **(A)** and Cave No. 256 of the Mogao Grottoes **(B)** for entire year. Six monitoring sites of the Maijishan Grottoes comprising wall painting plaster layer of Cave No. 28 and Cave No. 30 (28N-IN, 30N-IN), wall painting surface of Cave No. 28 and Cave No. 30 (28N-S, 30N-S), air of Cave No. 28 and Cave No. 30 (28-3, 30N-3); Five monitoring sites of the Mogao Grottoes comprising one for air at the eastern part of the southern slope (ESA) and four for murals at the north part of the northeast roof (NER), the west wall (WW), the eastern part of southern roof (ESR), and the northwest wall (NWW).

RH data showed that the mean diurnal RH range at all plaster layers monitoring points in both caves of Maijishan Grottoes (2% ~ 10%) exceeded those in Mogao Grottoes Cave No. 256 of (1% ~ 5%). In Maijishan, Cave No. 30 exhibited the highest RH fluctuation in cave air (30-3), peaking at 23.32% in December, while its plaster layer (30 N-IN) remained relatively stable humidity (Figure 7A). Both caves showed higher daily RH variations on wall painting surfaces than in plaster support layers, suggesting that surface RH is more sensitive to ambient changes. At Cave No. 256 of Mogao Grottoes, RH in cave air showed strong seasonal variation associated with precipitation (Figure 7B), with diurnal fluctuations ranging from 2.11% (February) to 9.80% (July). From February to October, RH fluctuations in cave air exceeded those in plaster layers. The west wall (WW) plaster layer displayed exceptional RH stability, with fluctuations remaining below 2%, likely due to its location far from the cave entrance and limited air exchange (Figure 7B).

Daily maximum RH frequency analysis showed that in Maijishan Grottoes (Caves No. 28 and 30), RH most often fell within 70–80% at all monitoring points (Table 2). In Cave No. 28, RH > 70% occurred on 214 days at the plaster layer (28 N-IN), 207 days at the wall painting surface (28 N-S), and 185 days in cave air (28-3). In Cave No. 30, corresponding values were 209 days (30 N-IN), 202 days (30 N-S), and 146 days (30-3), respectively. Extreme RH events (>90%) were more common in Cave 28 and more frequent in plaster layers than on wall painting surfaces. In contrast, at Mogao Cave No. 256, daily maximum RH at most points fell within 20–30%, with RH > 70% occurring only on a few days, and never exceeding 80% (Table 2). Notably, the west wall (WW), showing visible water damage, had 73 days with RH > 60%, much higher than other plaster sites. The northeast roof (NER) near the entrance remained RH < 60% year-round.

**Table 2:** Daily maximum relative humidity distribution frequency of monitoring sites in investigative caves of two heritage sites.

Relative humidity (%)	Monitoring sites										
	Cave No. 256 of the Mogao Grottoes					Cave No. 28 and 30 of the Maijishan Grottoes					
	ESA	NER	WW	ESR	NWW	28 N-IN	28 N-S	28-3	30 N-IN	30 N-S	30-3
0–10	0	0	0	0	0	0	0	0	0	0	0
10–20	75	1	0	87	65	0	0	0	0	0	0
20–30	119	189	130	97	105	0	0	1	0	0	2
30–40	64	90	70	69	68	13	23	26	8	23	25
40–50	45	55	62	45	42	33	28	28	45	32	34
50–60	32	30	30	34	45	56	48	71	72	61	66
60–70	25	0	65	32	33	49	59	54	31	47	92
70–80	5	0	8	1	7	84	78	81	83	84	102
80–90	0	0	0	0	0	73	74	65	65	69	43
90–100	0	0	0	0	0	57	55	39	61	49	1

Six monitoring sites of Maijishan Grottoes comprising wall painting plaster layer of Cave No. 28 and Cave No. 30 (28 N-IN, 30 N-IN), wall painting surface of Cave No. 28 and Cave No. 30 (28 N-S, 30 N-S), air of Cave No. 28 and Cave No. 30 (28–3,30 N-3); Five monitoring sites of the Mogao Grottoes comprising one for air at the eastern part of the southern slope (ESA) and four for wall painting at the north part of the eastern slope (NER), the west wall (WW), the eastern part of southern slope (ESR), and the northwest wall (NWW).

## 4 Discussion

### 4.1 The core taxa and their biodeterioration potential in Maijishan Grottoes

In this study, combined DNA-based and RNA-based Illumina MiSeq sequencing was employed to comprehensively characterize both total and metabolically active bacterial communities associated with black and white mycelial biofilms in the Maijishan Grottoes. RNA-level sequencing enabled insight into microbial activity, complementing DNA-based community assessments and offering a more dynamic perspective on active biodegradation risks (Meng et al., 2020; Li et al., 2021). Bacterial *α*-diversity and overall taxonomic profiles showed no significant differences between black and white mycelia within Maijishan Grottoes, suggesting that community composition alone cannot account for the observed phenotypic color variation (Langille et al., 2013; Quince et al., 2017). The distinct coloration of black and white mycelia might arise from a combination of microbial communities, pigment production (e.g., melanins, carotenoids), and the physicochemical properties of extracellular polymeric substances (EPS, Hathaway et al., 2014; Qin and Xia, 2024; Zomer et al., 2024). Moreover, mycelia sampled from the same cave shared highly similar bacterial communities regardless of their color, indicating the dominant influence of cave-specific microenvironmental conditions, and supporting that microenvironmental parameters are the primary determinants of bacterial community assembly (Lavoie et al., 2017).

Microbial biofilms across diverse cultural heritage sites often shared microbial phyla (Hathaway et al., 2014; Duan et al., 2021). Regarding bacterial community structures, Actinobacteria dominated most samples in both DNA and RNA samples (except for sample B1R). Actinobacteria and Proteobacteria are typical phyla have been widely documented in heritage environments such as caves or subterranean sites (Jurado et al., 2005, 2008; Li et al., 2024). As prolific producers of secondary metabolite producers, Actinobacteria contribute to wall painting deterioration by releasing pigments, acids, and antimicrobial compounds, thereby promoting chromatic biofilm formation (Lepinay et al., 2018; Oliveira et al., 2017; Wang et al., 2012). Proteobacteria serve as keystone chemoorganotrophic degraders, functioning as sensitive biological indicators of active biodeterioration (Ai et al., 2022; Khadka et al., 2017).

At the genus level, *Pseudonocardia* was particularly dominant in samples from Cave No. 30, corroborating its established role as an important taxon in wall paintings at the Maijishan Grottoes (Duan et al., 2017). This genus exhibited acidogenic metabolism capable of modulating local pH to favor oligotrophic proliferation, a critical adaptation enabling dominance in xeric wall painting substrates (Duan et al., 2017; Stomeo et al., 2008). *Pseudonocardia* thrived in subterranean heritage sites under harsh conditions, such as low light, scarce organic matter, high salinity, elevated humidity and temperature (Laiz et al., 1999), their rapid growth could lead to the formation of white spots on wall paintings (Duan et al., 2021). Another dominant genus, *Actinomycetospora*, was implicated in microbial outbreaks across multiple caves of the Maijishan Grottoes (He et al., 2021). Importantly, RNA sequencing revealed that *Escherichia-Shigella* and *Stenotrophomonas* were substantially more abundant at the RNA-level than in DNA profiles, indicating high metabolic activity despite their relatively lower overall abundance. Thereinto, *Escherichia-Shigella* (Proteobacteria) often associated with intestinal microbiota (Stoddard et al., 1998; Wang et al., 2013), likely originate from animal vectors such as rodents, birds, or arthropods inhabiting the forested surroundings of the Maijishan Grottoes. These animals are likely to act as vectors for microbial dispersal, introducing exogenous microorganisms (e.g., intestinal microbiota via excreta) into the cave ecosystem. Their activity may exacerbate biodeterioration risks by altering microhabitat conditions or facilitating organic matter accumulation, thereby fostering microbial proliferation on wall paintings (Liu W. et al., 2022; Bastian et al., 2010). Additionally, *Rubrobacter* was identified in samples, aligning with findings from the Angkor Thom Bayon Temple (Zhang et al., 2018) and other caves of the Maijishan Grottoes (Duan et al., 2017; He et al., 2021), which was recognized as a core genus associated with rosy discoloration on wall paintings, sandstone, and masonry (Schabereiter-Gurtner et al., 2001; Imperi et al., 2007; Nugari et al., 2009). Together, the RNA results demonstrate that a subset of the bacterial community is actively engaged in processes with direct biodeterioration potential, highlighting the importance of integrating RNA data into conservation-oriented microbial risk assessments.

Under favorable growth conditions, microorganisms can colonize on wall paintings by utilizing carbon sources, nitrogen sources, and mineral nutrients derived from original painting materials and restoration plasters (Meng et al., 2020). In this study, several differences were observed in total organic carbon, total nitrogen, and electrical conductivity between Cave No. 28 and Cave No. 30. These differences might alter microenvironmental conditions (Li and Gu, 2022) and provide distinct nutrient substrates for microbial proliferation (Imperi et al., 2007; Sterflinger and Pinzari, 2012). Combining with cave-specific temperature and humidity, microenvironmental conditions might lead the differences in bacterial community composition between the two caves.

### 4.2 Difference of bacterial community and their adaptions

Across both sites, Actinobacteria, Proteobacteria, and Firmicutes were the core bacterial phyla associated with biodeterioration, consistent with their recognized roles as pioneer colonizers across diverse lithic heritage substrates (Li et al., 2017; Liu et al., 2019; Xing et al., 2023). Actinobacteria was the dominant bacterial phylum at two sites of this study. However, the relative abundance of Actinobacteria in Mogao samples was markedly lower in the Maijishan Grottoes samples. The Maijishan Grottoes exhibited sustained high humidity levels year-round, with extreme humidity events exceeding 90%, which are mainly attributed to their unique geographical setting characterized by dense forest coverage, frequent precipitation, and limited cave ventilation (Yao et al., 2022). The observed variation in Actinobacteria abundance may originate from their hydrophilic properties, the persistently humid microenvironment promoted actinobacterial spore germination and growth (Zvyagintsev et al., 2007). Conversely, samples from the Mogao Grottoes exhibited a higher relative abundance of Firmicutes. Certain members of the phylum Firmicutes, particularly within the classes Bacilli (e.g., *Bacillus*) and Clostridia (e.g., *Clostridium*), are capable of forming endospores, which serve as a key mechanism for surviving extreme environments such as drought. These endospores possess a multilayered protective structure that resists desiccation, high temperatures, and UV radiation, significantly enhancing their survival rates under arid conditions (Setlow, 2007; Mckenney et al., 2013). The arid climate of the Dunhuang region selected for the desiccation-resistant metabolic strategies of Firmicutes, enabling their ecological dominance in the Mogao Grottoes’ ecosystems (Ma et al., 2015). Proteobacteria emerged as the dominant taxa driving the biodeterioration processes on cultural heritage substrates, particularly Alpha-proteobacteria, played a pivotal role in heritage biodeterioration through their metabolic versatility and adaptive strategies. Part of Proteobacteria exhibited oligotrophic capabilities, enabling colonization of nutrient-poor stone surfaces via the utilization of diverse organic compounds and UV-resistant pigment production (Pinhassi and Berman, 2003; Yu et al., 2024). At the genus level, *Rhodococcus* was dominant in samples of the Mogao Grottoes, they were also frequently documented in global heritage biodeterioration cases. Conversely, *Pseudonocardia* dominated the samples of Maijishan Grottoes, a genus commonly associated with wall paintings in caves and tombs (Stomeo et al., 2008; Portillo et al., 2009; Liu W. et al., 2022). Several studies have confirmed that Actinobacteria (e.g., *Pseudonocardia*) exhibit strong adaptability to oligotrophic conditions with limited organic nutrients. As pioneering colonizers in natural caves, they initiate early growth and establish material foundations for other microorganisms (Ciferri, 1999). Moreover, *Pseudonocardia* actively modifies environmental pH through metabolic processes, creating localized slightly alkaline microenvironments that promote rapid proliferation, then contributed to biodeterioration (Portillo and Gonzalez, 2011).

Only 13 OTUs belonged to few overlapping bacterial genera were identified between the two sites, revealing the possible role of site-specific environmental conditions (e.g., humidity, temperature, and substrate composition) in shaping bacterial community composition on wall paintings, the microenvironment exerting selective pressure on bacterial communities (Allen et al., 2020). Microbial damages to wall paintings were typically shaped by the metabolic activities and successional dynamics of diverse microbial communities, comprising bacteria, fungi, and extracellular polymeric substances (EPS). Studies indicated that bacteria play critical roles in microbial biofilms as ecological modulators, dynamically reshaping the composition of other biofilm-associated microorganisms (e.g., fungi, algae) in response to microenvironmental fluctuations (Liu et al., 2020). In our study, bacteria likely serve analogous functions: though not dominant in white/black mycelia, they might respond to microenvironmental variations and influence these mycelia on wall paintings.

### 4.3 Interplay between environments and bacterial communities

The Maijishan Grottoes, situated in a forested mountainous region with persistent high humidity, the temperature and RH fluctuations might be predominantly influenced by seasonal changes and heat exchange of cliff rock. The Cave No. 28 and Cave No. 30 maintained RH > 70% for over half the year, creating ideal conditions for microbial outbreaks and biodeterioration (He et al., 2022). Rainfall infiltration through cliff fractures and monsoon-driven precipitation (July–September) elevate RH to near 90% for >50 days annually, triggering efflorescence, cracking, pigment layer exfoliation and plaster layers destabilization via thermal expansion-contraction cycles (Bertolin et al., 2020; Li and Gu, 2022; Wang et al., 2025; Rivera et al., 2006). Despite high microbial risk in the Maijishan Grottoes, the bacterial and fungal concentrations (mean: 754 CFU/m^3^ and 645 CFU/m^3^, respectively) maintained at a lower level (Duan et al., 2018). Air microbial concentrations between 500 and 1,000 CFU/m^3^ indicated moderate visitor impact, belonging to balanced level, necessitating moderate crowd control (Porca et al., 2011).

The Mogao Grottoes, located in a desert region with distinct seasons, experienced extreme aridity and significant diurnal temperature fluctuations. Cave No. 256 maintained a relatively lower humidity, with RH consistently below 70%. This stable microenvironment can inhibit microbial colonization, favoring heritage preservation (Loli and Bertolin, 2018). In addition, this cave exhibited minimal daily temperature variation (diurnal range <1°C), which typically supports microbial proliferation, but the arid conditions (RH < 70% for over two-thirds of the year) inhibited numerous microbial growth (Spagnuolo et al., 2019; Sterflinger, 2010). Low precipitation, intense solar radiation, and high evaporation accelerated salt efflorescence on wall paintings via the migration of capillary water, leading efflorescence and then induced painting layers detachment. Microbial communities undergo niche selection on the wall paintings with efflorescence progression, the drought-adapted taxa potentially accelerating growth and deterioration (Ma et al., 2020).

In the Mogao Grottoes samples, more than half of the OTUs were exclusively detected in the two types (BS, NBS) of samples from this location, while at the Maijishan Grottoes, over one-third of the OTUs were shared across all four sample types (W-DNA, B-DNA, W-RNA, B-RNA). The bacterial communities demonstrated significant divergence in both diversity and structural composition at two sites, with community characteristics showing strong spatial dependency on sampling locations. The composition of microbial communities on cultural heritage surfaces are also shaped by dynamic interactions between substrate physicochemical properties (e.g., organic carbon content, nitrogen content, electric conductivity) and site-specific environmental stressors within the microenvironment such as pH, temperature and humidity (Chen and Gu, 2022; Dias et al., 2023; Ding et al., 2021; Duan et al., 2018, 2021; Zhang et al., 2018). Among these variables, environmental conditions emerged as an important determinant, critically modulating both microbial taxonomic dominance and their metabolic functionality across heritage ecosystems (Biagioli et al., 2024). Concurrently, increasing global tourism compounds risks through organic contamination and microclimatic destabilization (Ai et al., 2022; Li et al., 2022). In these semi-enclosed ecosystems, niche filtering overrides dispersal limitations (Saarela et al., 2004; Vellend, 2010). Climatic parameters critically modulate the ecological dominance of microbial taxa in heritage sites (Biagioli et al., 2024). Bacterial communities exhibited remarkable adaptability and resilience under different environmental stress, with their composition dynamically adjusting to maintain stability in response to fluctuant microenvironmental conditions (Chen et al., 2023).

## 5 Conclusion

This study revealed significant differences in bacterial communities and drivers of microbes on wall paintings in the Maijishan and Mogao Grottoes. Persistent high humidity and cliff seepage in the Maijishan Grottoes might promote Actinobacteria dominance (e.g., *Pseudonocardia*), their metabolic byproducts (organic acids, pigments) might exacerbate painting layer degradation. In contrast, the arid conditions of the Mogao Grottoes selected for desiccation-tolerant Firmicutes (e.g., *Bacillus*) and halophilic Proteobacteria (e.g., *Halomonas*), with salt efflorescence posing a primary threat to integrity of wall paintings. Therefore, site-specific microbial risks call for tailored conservation interventions. For the Maijishan Grottoes, strategies should prioritize humidity regulation (e.g., cyclic dehumidification) and control of Actinobacterial spore. In addition, RNA-level analysis identified metabolically active enteric bacteria (e.g., *Escherichia*) in the Maijishan Grottoes, potentially introduced by wildlife, thus more attention is necessary for the future preventative conservation. The lack of shared dominant genera between two heritage sites emphasizes the importance of localized microbial surveillance and environment-specific preservation approaches. These findings may advance our understanding of microbial ecology in cultural heritage environments and potentially support the development of evidence-based, sustainable conservation strategies for ancient wall paintings.

## Data Availability

The datasets presented in this study are publicly available. This data can be found here: https://www.ncbi.nlm.nih.gov/, accession number PRJNA950462.
