# Supplementary material for: Difference and environmental drivers of bacterial communities on wall paintings of the Maijishan and Mogao Grottoes, China
Source: Front Microbiol. 2025 Sep 24;16:1657118. doi: 10.3389/fmicb.2025.1657118 (PMC12504278; doi:10.3389/fmicb.2025.1657118)
Supplement: Supplementary file 1 [file Data_Sheet_1.DOCX]

**Difference and environmental drivers of bacterial communities on wall paintings of the Maijishan and Mogao Grottoes, China**

**Wenxia Ma*^a,c^*, Qiqi Chen*^c^*,** **Fasi Wu*^b*^*, Dongpeng He*^b,c^*, Yulong Duan*^d^*, Yongqiang Yue*^g^*, Ji-Dong Gu*^e,f^*, Xiaoyan Yang *^a^*,** **Huyuan Feng*^c*^***

^a^ Key Laboratory of Western China’s Environmental Systems (Ministry of Education), Key Scientific Research Base of Bioarchaeology in Cold and Arid Regions (National Cultural Heritage Administration), College of Earth and Environmental Sciences, Lanzhou University, Lanzhou, Gansu, 730000, P.R. China.

^b^ National Research Center for Conservation of Ancient Wall Paintings and Earthen Sites, Conservation Institute, Dunhuang Academy, Dunhuang, Gansu, 736200, P.R. China.

^c^ MOE Key Laboratory of Cell Activities and Stress Adaptations, School of Life Sciences, Lanzhou University, Lanzhou, Gansu, 730000, P.R. China.

^d^ Key Laboratory of Extreme Environmental Microbial Resources and Engineering, Gansu Province, Northwest Institute of Eco-Environment and Resources, Chinese Academy of Sciences, Lanzhou, Gansu 730000, P.R. China.

^e^ Environmental Science and Engineering Group, Guangdong Technion - Israel Institute of Technology, 241 Daxue Road, Shantou, Guangdong, 515063, P.R. China.

^f^ Guangdong Provincial Key Laboratory of Materials and Technologies for Energy Conversion, Guangdong Technion - Israel Institute of Technology, 241 Daxue Road, Shantou, Guangdong 515063, P.R. China.

^g^ Institute of Maijishan Grottoes Art, Dunhuang Academy, Tianshui, Gansu, 741020, P.R. China.

^*^**Corresponding:**

Fasi Wu

wufs@dha.ac.cn

Huyuan Feng

fenghy@lzu.edu.cn

**Supplementary Tables and Figures:**


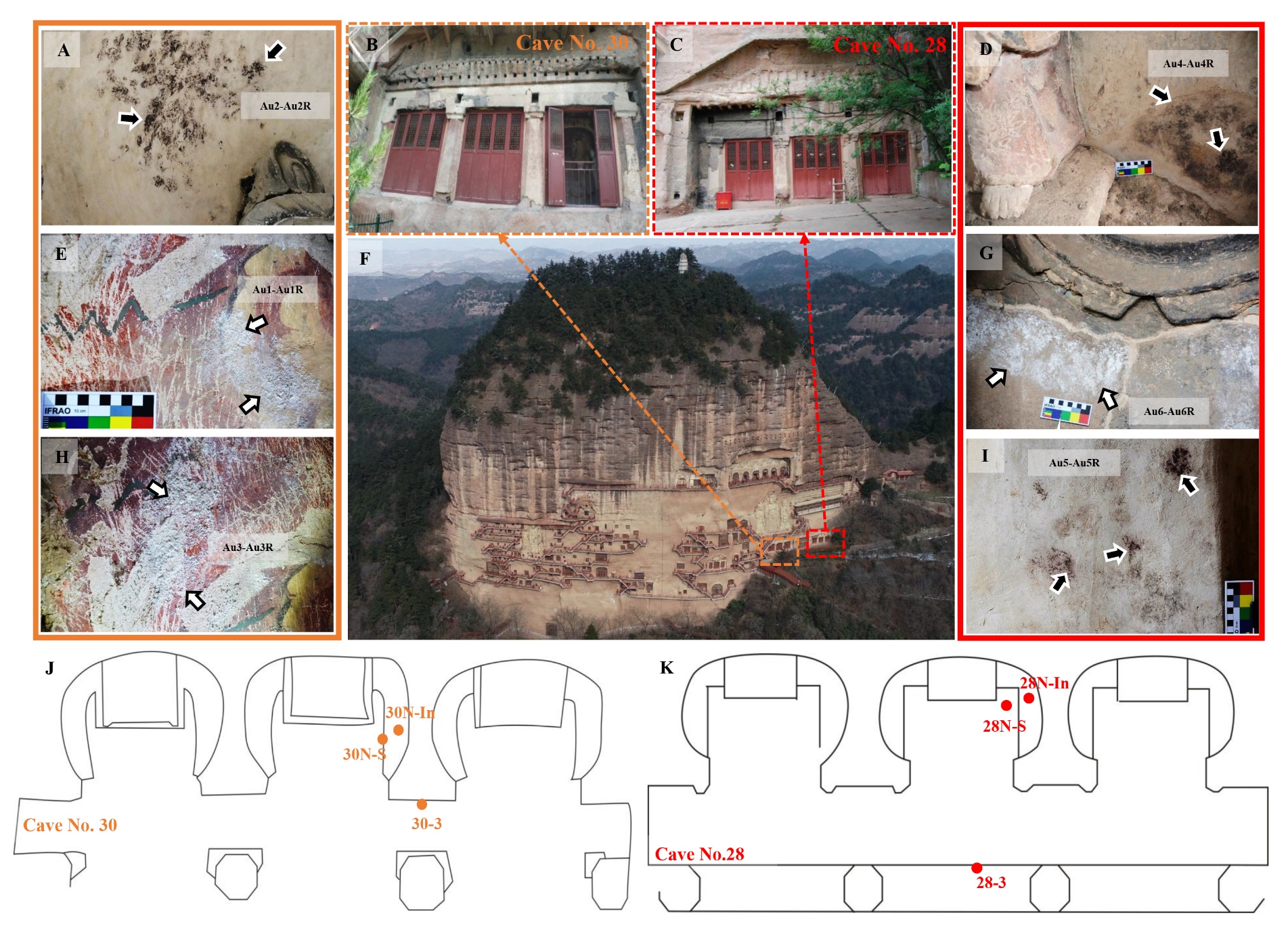


**Fig. S1.** Biofilms and sampling sites on wall paintings of Caves No. 28 and No. 30 in the Maijishan Grottoes. **(A, D, I)** Black biofilms colonized on the wall painting surfaces. **(B, C, F)** The exterior and location illustration of Cave No. 30 and Cave No. 28 in the Maijishan Grottoes. **(****E, G, H)** White biofilms colonized on the wall painting surfaces.


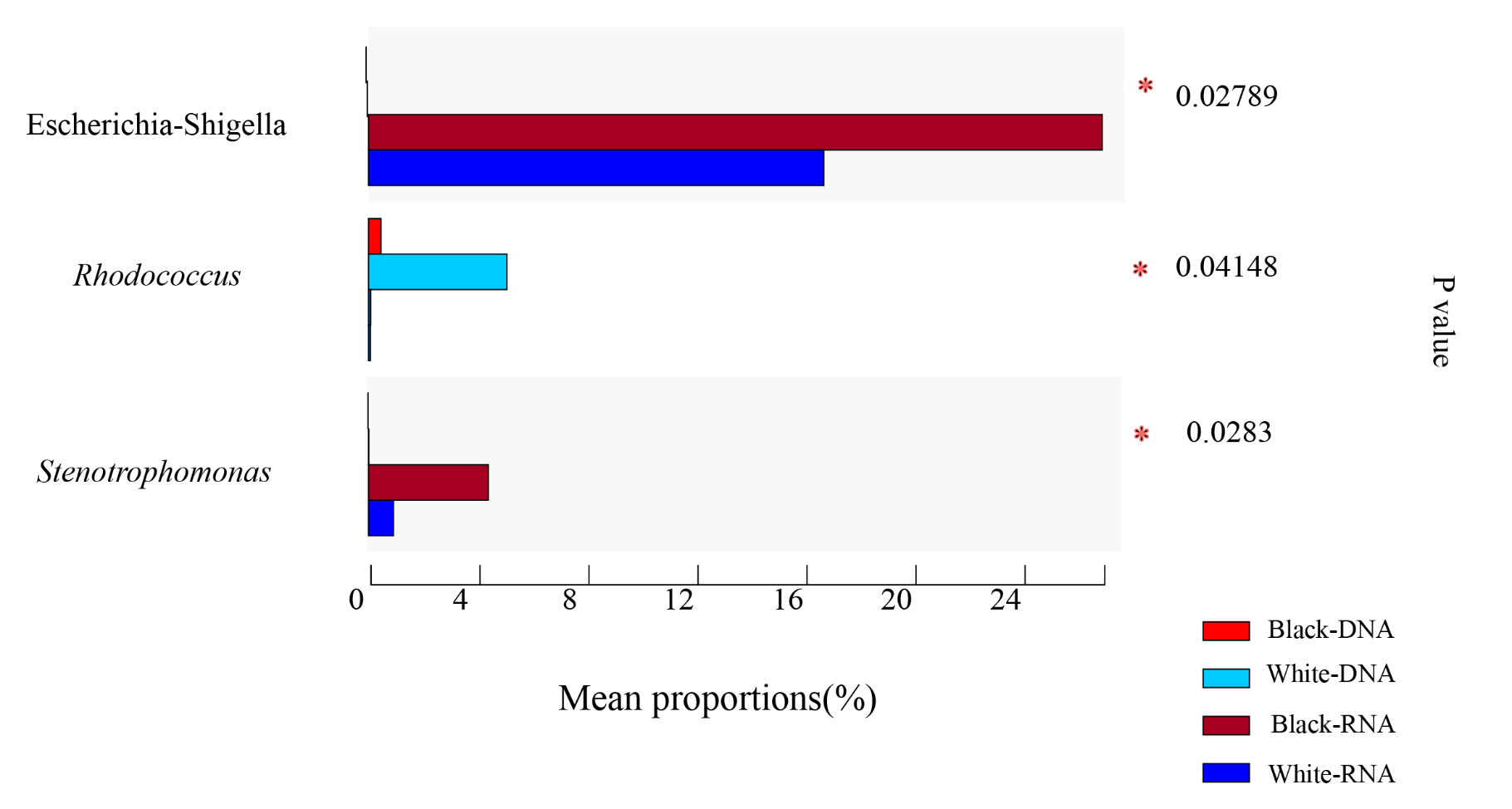


**Fig. S2.** Genus-level differences in bacterial abundances among the four groups (Black-DNA, White-DNA, Black-RNA, White-RNA) by Kruskal-Wallis H test.


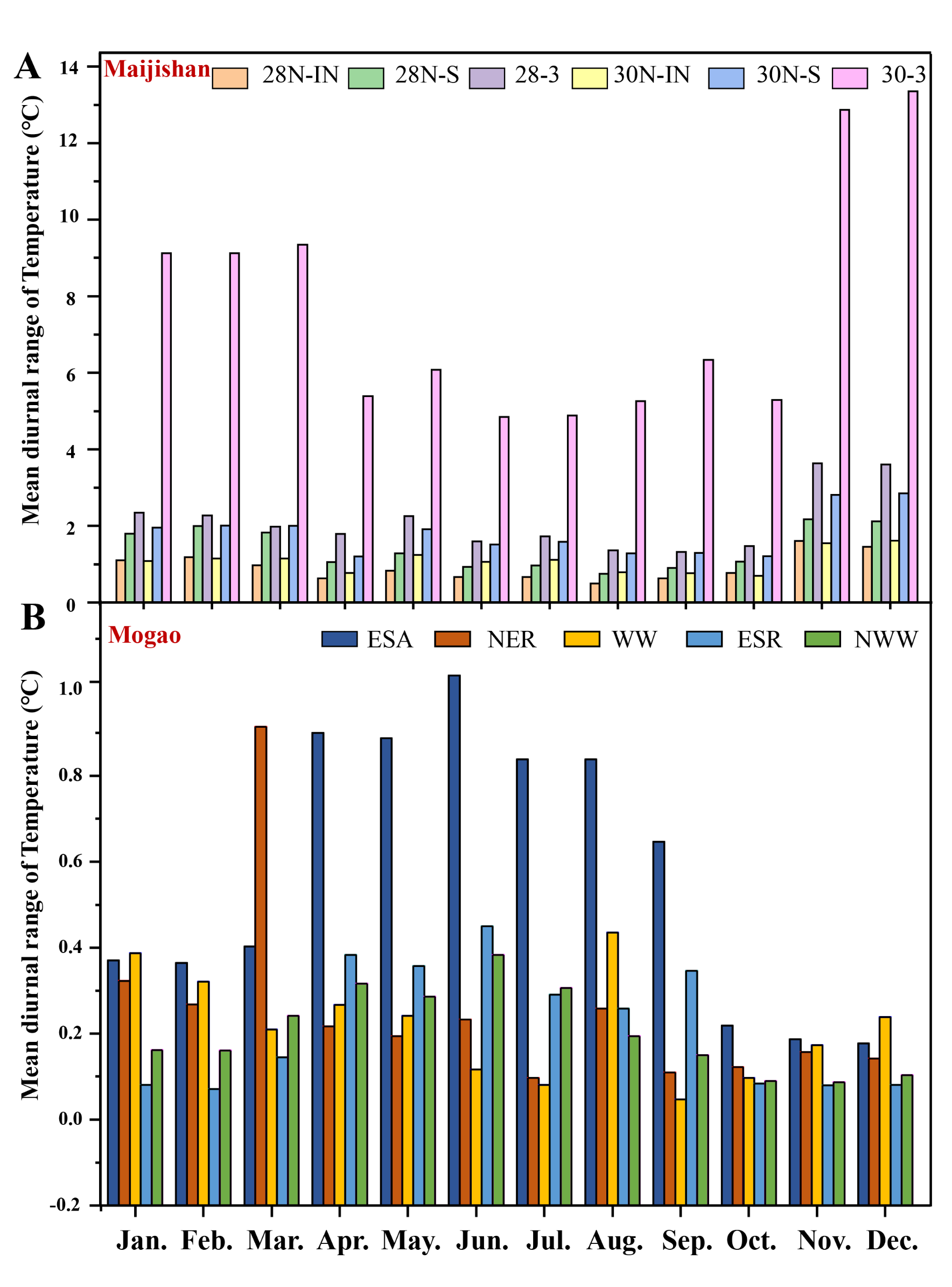


**Fig. S3.** Mean diurnal range of temperature within Caves No. 28 and No. 30 of the Maijishan Grottoes **(A)** and Cave No. 256 of the Mogao Grottoes **(B)** for entire year. Six monitoring sites of the Maijishan Grottoes comprising wall painting plaster layer of Cave No. 28 and Cave No. 30 (28N-IN, 30N-IN), wall painting surface of Cave No. 28 and Cave No. 30 (28N-S, 30N-S), air of Cave No. 28 and Cave No. 30 (28-3,30N-3); Five monitoring sites of the Mogao Grottoes comprising one for air at the eastern part of the southern slope (ESA) and four for murals at the north part of the northeast roof (NER), the west wall (WW)**,** the eastern part of southern roof (ESR), and the northwest wall (NWW).

**Table S1.** PCR reaction system for 16S rRNA Gene amplification in samples from Maijishan Grottoes

| **Components** | **Volume (μL)** |
| --- | --- |
| 5×Fast Pfu Buffer | 4 |
| dNTPs（2.5 mM） | 4 |
| Primer（each 5 μM） | 0.8 |
| TransStart FastPfu Polymerase | 4 |
| Template DNA | 1 |
| dd H2O | 9.8 |

**Table S2.** Phylotype coverage and diversity estimation of the 16S rRNA gene libraries of the samples from the high-throughput sequencing analysis

| **Sample Types** | **Samples** | **Reads** | **OTUs** | **Ace** | **Chao** | **Shannon** | **Simpson** |
| --- | --- | --- | --- | --- | --- | --- | --- |
| **Black-DNA** | B1 | 76327 | 106 | 110.60 | 109.00 | 1.88 | 0.22 |
|  | B2 | 42303 | 198 | 248.98 | 247.71 | 2.75 | 0.18 |
|  | B3 | 41317 | 174 | 266.26 | 261.89 | 2.36 | 0.22 |
| **Black-RNA** | B1R | 45687 | 107 | 128.84 | 123.06 | 1.08 | 0.59 |
|  | B2R | 43106 | 164 | 198.10 | 202.00 | 2.91 | 0.12 |
|  | B3R | 39709 | 165 | 186.78 | 187.50 | 2.73 | 0.14 |
| **White-DNA** | W1 | 132694 | 326 | 342.86 | 316.63 | 2.27 | 0.16 |
|  | W2 | 127770 | 243 | 254.81 | 255.22 | 2.20 | 0.23 |
|  | W3 | 38444 | 213 | 215.33 | 208.62 | 2.75 | 0.11 |
| **White-RNA** | W1R | 41502 | 133 | 211.55 | 180.79 | 2.09 | 0.17 |
|  | W2R | 40260 | 92 | 197.30 | 143.00 | 1.59 | 0.36 |
|  | W3R | 40299 | 181 | 215.89 | 228.59 | 2.69 | 0.11 |

Notes: The operational taxonomic units (OTUs) were defined with 97% similarity threshold. Richness estimators (ACE and Chao), and diversity indices (Shannon and Simpson)
